# Heme Interferes With Complement Factor I-Dependent Regulation by Enhancing Alternative Pathway Activation

**DOI:** 10.3389/fimmu.2022.901876

**Published:** 2022-07-22

**Authors:** Alexandra Gerogianni, Jordan D. Dimitrov, Alessandra Zarantonello, Victoria Poillerat, Satheesh Chonat, Kerstin Sandholm, Karin E. McAdam, Kristina N. Ekdahl, Tom E. Mollnes, Camilla Mohlin, Lubka T. Roumenina, Per H. Nilsson

**Affiliations:** ^1^ Linnaeus Centre for Biomaterials Chemistry, Linnaeus University, Kalmar, Sweden; ^2^ Department of Chemistry and Biomedicine, Linnaeus University, Kalmar, Sweden; ^3^ Centre de Recherche des Cordeliers, INSERM, Sorbonne Université, Université de Paris, Paris, France; ^4^ Aflac Cancer and Blood Disorders Center, Children’s Healthcare of Atlanta, Atlanta, GA, United States; ^5^ Department of Pediatrics, Emory University School of Medicine, Atlanta, GA, United States; ^6^ Department of Immunology, Oslo University Hospital and University of Oslo, Oslo, Norway; ^7^ Department of Immunology, Genetics and Pathology, Rudbeck Laboratory, Uppsala University, Uppsala, Sweden; ^8^ Centre of Molecular Inflammation Research, and Department of Clinical and Molecular Medicine, Norwegian University of Science and Technology, Trondheim, Norway; ^9^ Research Laboratory, Nordland Hospital, Bodo, Norway

**Keywords:** heme, complement, factor I, co-factor activity, hemopexin, hemolysis

## Abstract

Hemolysis, as a result of disease or exposure to biomaterials, is characterized by excess amounts of cell-free heme intravascularly and consumption of the protective heme-scavenger proteins in plasma. The liberation of heme has been linked to the activation of inflammatory systems, including the complement system, through alternative pathway activation. Here, we investigated the impact of heme on the regulatory function of the complement system. Heme dose-dependently inhibited factor I-mediated degradation of soluble and surface-bound C3b, when incubated in plasma or buffer with complement regulatory proteins. Inhibition occurred with factor H and soluble complement receptor 1 as co-factors, and the mechanism was linked to the direct heme-interaction with factor I. The heme-scavenger protein hemopexin was the main contaminant in purified factor I preparations. This led us to identify that hemopexin formed a complex with factor I in normal human plasma. These complexes were significantly reduced during acute vasoocclusive pain crisis in patients with sickle cell disease, but the complexes were normalized at their baseline outpatient clinic visit. Hemopexin exposed a protective function of factor I activity *in vitro*, but only when it was present before the addition of heme. In conclusion, we present a mechanistic explanation of how heme promotes uncontrolled complement alternative pathway amplification by interfering with the regulatory capacity of factor I. Reduced levels of hemopexin and hemopexin-factor I complexes during an acute hemolytic crisis is a risk factor for heme-mediated factor I inhibition.

## Introduction

Intravascular hemolysis is observed in various diseases, including sickle cell disease (SCD) (1), malaria, atypical hemolytic uremic syndrome (2), and sepsis (3). Hemolysis can also be a complication after a blood transfusion or exposure to biomaterials, where mechanical forces in the cardiovascular devices can rupture erythrocytes (4–6). Upon disruption of the erythrocytes, hemoglobin is released into the plasma. Heme is an iron prosthetic group and an essential component of hemoglobin and other hemeproteins (7). Under pro-oxidative conditions, heme is oxidized into the ferric (Fe^3+^) state and is then prone to be released from the hemoglobin (7, 8). This plasma free heme is a pro-oxidative and pro-inflammatory mediator that can trigger innate immune activation (9). Studies have linked heme to innate immune activation *via* triggering complement- and Toll-like receptor 4-activation (10, 11). Platelets, endothelial cells (12, 13), and the coagulation system (14, 15) are other vasculature components that heme can activate.

Any toxicity from hemoglobin and heme is compensated by the presence of abundant plasma scavenger molecules (16). Haptoglobin, an acute-phase protein, captures hemoglobin and prevents heme from being released. Monocytes express CD163 that clear haptoglobin-hemoglobin from plasma, and endothelial cells can induce expression of heme-oxygenases to degrade heme into less toxic byproducts. Additionally, plasma hemopexin binds plasma-free heme and neutralizes its reactivity (4, 8, 17–19). However, these systems can be saturated under massive or continuous hemolysis (20).

Activation of the complement system can occur *via* three routes: classical, lectin, and the alternative pathway (21). All three pathways lead to the formation of a convertase that cleaves C3 into C3a and C3b. Every surface-bound C3b can initiate or propagate the alternative pathway activation, thus, rapidly amplifying the response. Therefore, it is critical to immediately degrade C3b into iC3b to control this amplification course on non-pathogenic substrates and host surfaces. C3b is degraded by factor I to iC3b and C3f, and iC3b can further be degraded to C3c and C3dg fragments (22–24). Factor I is a serine protease that requires the presence of co-factors, i.e., the soluble factor H, the membrane-bound complement receptor 1 (CR1, CD35), or the membrane co-factor protein (MCP, CD46), to cleave the alpha´-chain of C3b (25).

Multiple studies have linked intravascular hemolysis and the presence of cell-free heme in plasma to alternative complement pathway activation (26–29). The mechanism is not fully understood but is related in part to the interaction of heme with C3 (2). Among the regulators of C3, factor H shows weak interaction with heme, but no data are available for factor I (2). This study aimed to investigate how heme dysregulates complement regulatory function at the level of factor I.

## Materials and Methods

### Reagents

Porcine hemin [ferriprotoporphyrin IX chloride] obtained from Merck (Darmstadt, Germany) was prepared as previously described (2). Hemin refers to the oxidized Fe^3+^-analog of heme. Human factor C3 was purified according to the method described by Hammer et al. (30). C3b was prepared by digestion of C3 with 1% (w/w) trypsin from bovine pancreas (Merck) for 5 minutes at 22°C and separated with gel filtration on Sephadex G100 (GE Healthcare, Chicago, IL), which was then equilibrated with 10 mM phosphate buffer (pH 7.4) and 0.145 M saline (PBS). Human complement factor H was purified from human serum as described by Hammer et al. (30) but modified with euglobulin precipitation, according to Nilsson et al. (31). Human complement factor I was procured from CompTech (Tyler, TX) and human plasma hemopexin from Merck. Biotinylated antibodies were prepared with the biotinylation reagent biotinamidohexanoic acid N-hydroxysuccinimide ester (Merck) (32). The compstatin analog Cp40 [yI[CV(MeW)QDW-Sar-AHRC](NMe)I-NH_2_] to block C3-cleavage (33), was kindly provided by John D. Lambris. Soluble CR1 (sCR1) was obtained from T Cell Sciences Inc (Cambridge, MA).

### Whole Blood Collection and Plasma Preparation

Patients with sickle cell disease were recruited at Children’s Healthcare of Atlanta, Georgia, USA, under a local IRB-approved study. Ethylenediaminetetraacetic acid (EDTA)-plasma samples were collected at two time points; hospital admission for acute vasoocclusive pain crisis (VOC) and during their baseline outpatient clinic at least four weeks later. Whole blood was collected from healthy donors (n = 20) in 4.5 mL cryotubes (Cryo Tube™ Vials; Thermo Scientific, Waltham, MA) containing thrombin inhibitor, lepirudin (Refludan; Celgene, Uxbridge, UK) at a final concentration of 50 μg/mL. Whole blood was centrifuged at 3000 x g for 15 minutes to collect plasma. A human plasma pool (NHP) was created by pooling plasma from six healthy donors. All plasma samples were stored at -80°C.

### Absorbance Spectroscopy

Aliquots of heme, resulting in final concentrations of 0.25 to 64 μM, were added to an optical cell containing factor I (CompTech) or PBS as control, and incubated for two minutes in the dark at room temperature. The absorbance spectra in the 350 – 700 nm wavelength range were recorded using an Agilent Cary 100 spectrophotometer (Agilent Technologies, Santa Clara, CA). The data on the difference between protein-bound heme and free heme at absorbance maxima (λ = 395 nm) in the Soret peak was used to build titration binding curves.

### Measurement of Binding of Heme to Factor I by Surface Plasmon Resonance

The binding of heme to factor I was further evaluated by surface plasmon resonance (BIAcore 2000 instrument). Factor I (CompTech) was immobilized on a CM5 sensor chip using an amino-coupling kit as recommended by the manufacturer (Cytiva, Uppsala, Sweden), and a control flowcell was mock-immobilized. The experiments were performed using PBS (pH 7.4) with 0.005% (v/v) Tween 20 as a running buffer. Heme was diluted from a 10 mM stock in PBS just before injection and introduced to the flowcells at a flow rate of 10 μl/min. Regeneration of the chip surface was achieved by brief exposure (30 s) to a solution containing 0.3 M imidazole. The binding to the surface of the control flow cell was subtracted from the binding to the factor I-coated flow cells. BIAevaluation software (version 4.1; Biacore) was used to evaluate the kinetic rate constants.

### *In Silico* Analyses of Heme Binding to Factor I

The atomic coordinates of factor I were taken either from PDB ID 2XRC (34) or extracted from the C3b/fH1-4:19-20/FI (PDB ID 5O32) and used for the molecular docking (35). HexServer (http://hexserver.loria.fr/) with default parameters was used to accommodate the heme molecule to FI (36), as described previously for C1q and C3 (2, 37). Criteria for final factor I-heme complex selection were based on the total energy of binding. Visualization of the top ten complexes was done by PyMol (www.pymol.org). To evaluate for potential functional relevance, we visualized the predicted top ten heme-binding positions at the structure of FI alone or at the C3b/fH1-4:19-20/FI complex structure.

### Evaluation of Degradation of Soluble C3b

A complement co-factor assay was employed to determine C3b-degradation. C3b (10 μg) was incubated with factor I (0.1 μg) and the co-factors (0.1 μg) factor H or sCR1 in PBS for up to 60 minutes. Heme was included in the incubations in concentrations between 8 and 64 μM. Incubations that contained C3b, factor I, and factor H or sCR1 served as positive controls. Incubations that contained C3b, factor H, and sCR1, without factor I, served as negative controls. Hemopexin (40 μg/ml) was included during the incubation in separate experiments. In these setups, all samples contained heme at 32 μM. Four different 30-minute incubations were included for each of the two co-factors, i) all components (C3b, factor I, heme, hemopexin, and co-factor) were added simultaneously, ii) factor I was pre-incubated with heme for 15 minutes before addition of C3b, co-factor, and hemopexin, iii) all components but hemopexin were pre-incubated for 15 minutes before addition of hemopexin, iv) factor I was preincubated with hemopexin for 15 minutes before C3b, co-factor and heme were later added. The 30-minute incubation time started after adding all the components mentioned above. All incubations were carried out in 1.8 mL Nunc-cryotubes (Nunc, Roskilde, Denmark) at 37°C. Reduced (5x) SDS-PAGE sample buffer (Biorad, Hercules, CA) was added at the end of each incubation, and the samples were moved to a heating block and incubated for another 10 minutes at 95^°^C. Finally, the samples were loaded on 4 – 15% Tris-glycine gels and applied to SDS-PAGE electrophoresis. The gels were stained with Coomassie Brilliant Blue G-250 dye (Biorad).

### Quantification of Degradation of Surface-Bound C3b

According to the manufacturer’s protocol, Bio-Plex Pro™ magnetic COOH beads were immobilized with 6 μg cystamine sulfate hydrate (Merck) using the amine coupling kit (Biorad). Dithiothreitol (DTT; Roche Diagnostics, Rotkreuz, Switzerland) 6 mM was used for the reduction of the internal disulfide bond of cystamine (38). The beads were incubated with DTT at 22°C for 20 minutes, followed by thorough washing with PBS. To conjugate C3b to the free sulfhydryl of the immobilized cystamine, 20 μg of purified C3 and 1.6% (w/v) of trypsin were incubated with the beads for 20 minutes at 37°C. The reaction was later stopped by washing with PBS. The C3b-beads were then incubated in black flat-bottom 96-well plates together with factor I (0.15 μg) and factor H or sCR1 (1.3 μg), with increasing heme concentrations in three-fold serial dilutions (up to 32.4 μM). Trypsin (140 μg/mL) incubated with C3b-beads was used as a positive control. After incubation for one hour at 37°C on agitation, C3b-fragments were detected using two different antibodies, either biotinylated polyclonal rabbit anti-human C3c antibody (Dako, 4 μg/mL) or biotinylated monoclonal anti-human-iC3b-neo (Quidel, A209, 4 μg/mL). Both antibodies were followed by streptavidin-phycoerythrin (PE; Biorad), diluted at 1:100. Incubations with the antibody and streptavidin took place for 30 minutes at 22°C on agitation in the dark. All dilutions were done in PBS with 0.1% (v/v) Tween 20 and 0.05% (w/v) bovine serum albumin (BSA) (Merck). The beads were washed three times with PBS 0.05% (v/v) Tween 20 between every incubation step. Mean fluorescent intensity (MFI) was detected by Bio-Plex MAGPIX Multiplex Reader (Biorad).

Likewise, we repeated this process with lepirudin-anticoagulant plasma instead of purified components. The plasma was then centrifuged for 10 minutes at 9400 x g and diluted 1:5 with PBS containing Cp40 (8 μM). Next, heme was added to the plasma in concentrations of up to 32.4 μM, and C3b-beads were added and incubated for up to 60 minutes at 37°C.

### Factor I Purification

Normal human citrate-plasma (235 mL) was supplemented with EDTA to 5 mM final concentration. Polyethyleneglycol (4 kDa) was added to the plasma at 8% (w/v) concentration, incubated for 30 minutes at 4°C on rotation, and then centrifuged at 13000 x g for 30 minutes at 4°C. The supernatant was isolated and diluted at 1:2 with 5 mM EDTA, and the pH was adjusted to 6.0 with hydrochloric acid (HCl). The supernatant was loaded on a 50 mL SP-Sepharose Fast Flow 26/10 50 mL column equilibrated with a 20 mM phosphate buffer (pH 6.0) with 50 mM sodium chloride (NaCl), 5 mM EDTA, and 0.02% (w/v) NaN_3_, with a flow rate of 5 mL per minute. The column was washed with 10 volume fractions of equilibration buffer and eluted with a 250 mL NaCl gradient (50 mM – 400 mM). One hundred 2.5 mL fractions were collected.

Factor I was detected in eluted fractions using direct ELISA. The fractions were coated in wells of a microtiter plate, 50 μl per well, and incubated overnight at 4°C. The wells were blocked with 1% (w/v) bovine serum albumin diluted in a 10 mM phosphate buffer containing 145 mM NaCl. Factor I was detected using sheep anti-human factor I (The Binding Site, Birmingham, UK), followed by a rabbit anti-sheep IgG with horseradish peroxidase (HRP) by Dako (Glostrup, Denmark). The fractions containing factor I (in total 130 mL) were pooled and dialyzed three times against 1 liter 10 mM Tris-HCl (pH 8.5), 200 mM NaCl, 0.02% NaN_3_. The dialysate was loaded, at 1 mL per minute flowrate, on a 15-mL Wheat Germ Agglutinin Sepharose column equilibrated with a 10 mM Tris-HCl, 200 mM NaCl, 0.02% NaN_3_ pH 8.5. The same buffer was used for washing the column, five column volumes. Factor I was eluted with 10% (w/v) N-acetyl-D-glucosamine supplemented to the washing buffer in 90 fractions, 0.5 mL each. As detected with the direct ELISA, the fractions containing factor I (34 fractions) were pooled and dialyzed two times against 1 L 10 mM Tris-HCl (pH 8.5) with 0.02% NaN_3_. The dialysate was applied, with a flow rate of 2.5 mL per minute, on an 8-mL Mono Q column, equilibrated with the 10 mM Tris-HCl (pH 8.5) 0.02% NaN_3_, and washed with 24 column fractions of the equilibration buffer. The column was eluted with a 0-220 mM NaCl-linear gradient supplemented with the equilibration buffer for 60 minutes, followed by 10 minutes with the 220 mM-NaCl buffer. The factions were evaluated for factor I and hemopexin.

As for factor I, hemopexin was detected in a direct ELISA with fractions diluted at 1:250 in PBS. Hemopexin was detected using a rabbit anti-human hemopexin (Dako) followed by a polyclonal anti-rabbit IgG-HRP (Dako). Depending on when factor I was eluted in the chromatogram, fractions were pooled in one early pool, labeled as pool 1, and one late pool, referred to as pool 2. The presence of hemopexin and factor I in pool 1 and pool 2 were assessed by Western blot. The samples (10 μg) included two commercial factor I preparations from CompTech and Quidel for comparison, and two hemopexin preparations, one commercial (R&D Systems, Abingdon, UK) and one purified in-house (27). The samples were mixed with reduced and non-reduced Laemmli loading buffer (Biorad) and heated for 10 minutes at 94°C. The proteins were separated on a 4%-15% SDS-PAGE and transferred to a polyvinylidene difluoride (PVDF) membrane (Biorad). Hemopexin was detected by a biotinylated rabbit anti-human hemopexin antibody (Dako) followed by streptavidin-HRP (Biorad). A biotinylated sheep anti-human factor I (Abcam) followed by streptavidin-HRP (Biorad) was used to identify factor I. The bands were detected by chemiluminescence and imaged by Chemi Doc ™ MP Gel Imaging System (Biorad). Finally, a functional test was applied to evaluate the ability of the two pools to degrade C3b effectively. Purified C3b (10 μg) was incubated with all the different factor I-preparations (2 μg), as analyzed above with Western blotting, and factor H (2 μg) at 37°C for 60 minutes in PBS (pH 7.4). The samples were run on a 4%-15% SDS-PAGE under reduced conditions and were stained with Coomassie Brilliant Blue.

### Hemopexin, Factor I, and Factor I/Hemopexin-Complex Immunoassays

Hemopexin was quantified in plasma by ELISA as previously described (27). Factor I and the factor I/hemopexin-complexes were quantified in immunoassays developed on the xMAP-platform. Bio-Plex Pro™ magnetic COOH beads (Biorad) were immobilized with monoclonal mouse anti-human complement factor I antibodies (Abcam, Cambridge, U.K.) using the Bio-Plex Pro™ magnetic COOH beads amine coupling kit according to the manufacturer’s instructions (Biorad). For both factor I and factor I/hemopexin-complexes, beads amounting to 2500 per well in 96-well plates (Bio-Plex Pro™ black flat bottom plate; Biorad) were incubated with EDTA-plasma samples (1:500 for the factor I/hemopexin-complexes and 1:300 for factor I), for 30 minutes at RT on agitation. Biotinylated rabbit anti-human hemopexin antibody (Dako), diluted at 1:50, followed by streptavidin-PE (Biorad), diluted at 1:100, was used to detect the complexes. Biotinylated sheep anti-human factor I (Abcam) diluted at 1:300, followed by streptavidin-PE (Biorad), diluted at 1:100, was used to detect factor I. Pool 1, co-purified with hemopexin, was used as the standard for measuring factor I/hemopexin complexes. The international complement standard #1 (39) was used as the standard for factor I. We followed the same incubation conditions for the C3b quantification protocol as described above. MFI was detected by Bio-Plex MAGPIX Multiplex Reader (Biorad).

### Statistical Analysis

The SDS-PAGE data was analyzed by Image J (US National Institutes of Health, https://imagej.nih.gov/ij). The statistical analysis and absorbance spectroscopy calculations were performed using GraphPad Prism version 7.7 for Mac (San Diego, CA). Paired t-test was applied to compare two columns, and either ordinary/repeated measures with Dunnette’s multiple comparisons or non-parametric one-way ANOVA were used to compare multiple columns.

## Results

### Heme Interacts With Factor I

**Figure 1:**
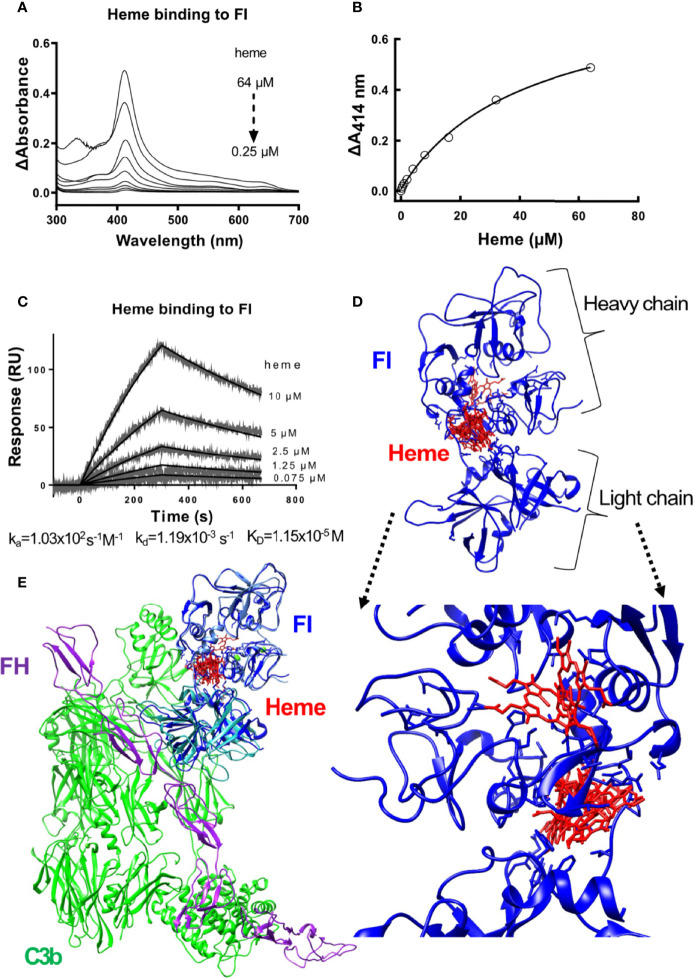
Heme binding to factor I **(A, B)** Absorbance spectroscopy of heme (0.25-64 μM) binding to factor I (FI) from Comptech, shown as an absorbance spectrum **(A)** and at wavelength 414 nm **(B)**. **(C)** SPR analysis of the binding of different concentrations of heme to factor I (Comptech) immobilized on a Biacore sensor chip. **(D)** Molecular docking of heme (red) to factor I (blue). The top ten complexes are visualized. **(E)** Overlap of the factor I-heme complexes with the structure of the complex C3b/fH1-4:19-20/FI (5O32).

Absorbance spectroscopy and surface plasmon resonance (SPR) were used to examine the ability of heme to interact with factor I. The absorbance spectra showed that heme bound to soluble factor I in a dose-dependent manner (**Figures 1A, B**). Increasing heme concentrations, up to 64 µM, increased the absorbance at 414 nm, referring to the Soret peak of heme. The interaction was dose-dependent but the curve did not reach saturation in our experimental conditions, to allow us to correctly deduce the binding affinity (an approximation from the fit would be ~40 µM). Therefore, we confirmed the binding by SPR. Heme at 0.075 to 10 µM was let to interact with surface-immobilized factor I, and the interaction was analyzed using SPR. Heme interacted with factor I with an apparent association rate (k_a_) of 1.03x10^2^ M^-1^s^-1^ and a dissociation rate (k_d_) of 1.19x10^-3^ s^-1^ (**Figure 1C**). The apparent equilibrium dissociation constant (K_D_) was calculated to 1.15x10^-5^ M (11.5 µM). *In silico* modeling was employed to predict the putative binding site between heme and factor I. The modelling indicated that the putative site of interaction of heme to factor I (both when taken alone (2XRC) or extracted from the C3b/fH1-4:19-20/FI (5O32)) was at the interface between the heavy and the light chain of factor I (**Supplementary Figure 1** and **Figure 1D**), and may potentially affect its enzymatic activity and the integration with the C3b/factor H and by homology, the C3b/CR1 complex (**Figure 1E**).

### Heme Inhibits Factor I-Mediated Degradation of Soluble C3b

**Figure 2:**
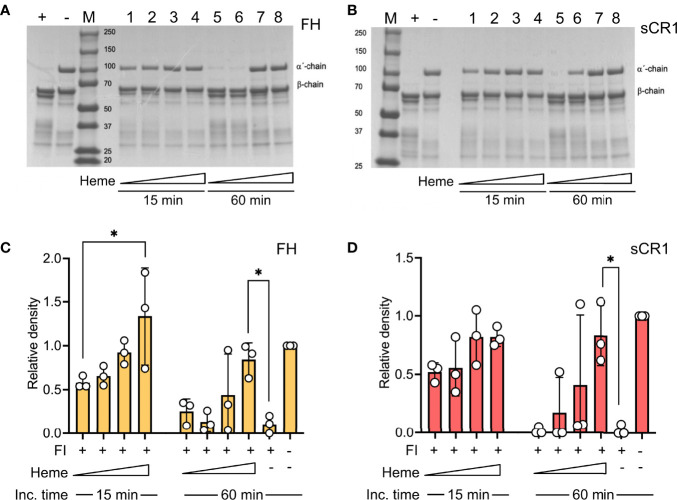
Effect of heme on factor I-mediated degradation of soluble C3b. C3b (10 μg) was incubated in the presence of factor I (FI, 0.1 μg) and **(A, C)** factor H (FH, 0.1 μg) or **(B, D)** soluble complement receptor 1 (sCR1, 0.1 μg) at 37°C for 15 and 60 minutes, in phosphate buffered saline (PBS) pH 7.4. The samples were run on a 4-15% SDS-PAGE under reduced conditions and stained with Coomassie Brilliant Blue. The gels shown **(A, B)** are each a representative gel from three independent experiments. **(A, B)** The samples are displayed as follows: positive control (+); negative control, which lacks FI (-); molecular weight marker (M). For samples 1-8, heme was included in increasing concentrations (lane 1, 5: 8 μM; lane 2, 6: 16 μM, lane 3, 7: 32 μM, lane 4, 8: 64 μM) for 15 minutes (1-4) or 60 minutes (5-8), as indicated in the figure. **(C, D)** C3b-degradation was evaluated by densitometric measurement and displayed as the relative density of the C3b intact α´-chain at 101 kDa in relation to the β-chain at 75 kDa. The values are shown as mean +/- standard deviation of n=3. *p < 0.05.

The impact of heme to factor I binding was investigated by evaluating factor I-mediated degradation of soluble C3b in the presence of heme. Purified C3b was incubated together with factor I and the co-factors factor H or soluble CR1 (sCR1) in increasing heme concentrations (8-64 μM) at 37°C. C3b-degradation was analyzed on an SDS-PAGE by densitometric comparison of the factor I-sensitive alpha´-chain (101 kDa) in relation to the inert beta-chain (75 kDa). Factor I degraded C3b in the presence of both factor H (**Figures 2A, C**) and sCR1 (**Figures 2B, D**). Heme interfered with this degradation; there was decreased cleavage of the α-chain of C3b as the heme concentration increased, both at 15- and 60-minutes incubation time. Heme at 64 μM significantly inhibited the C3b-cleavage, both with factor H and sCR1 as co-factors (p<0.05).

### Heme Inhibits Factor I-Mediated Degradation of Surface Bound C3b

**Figure 3:**
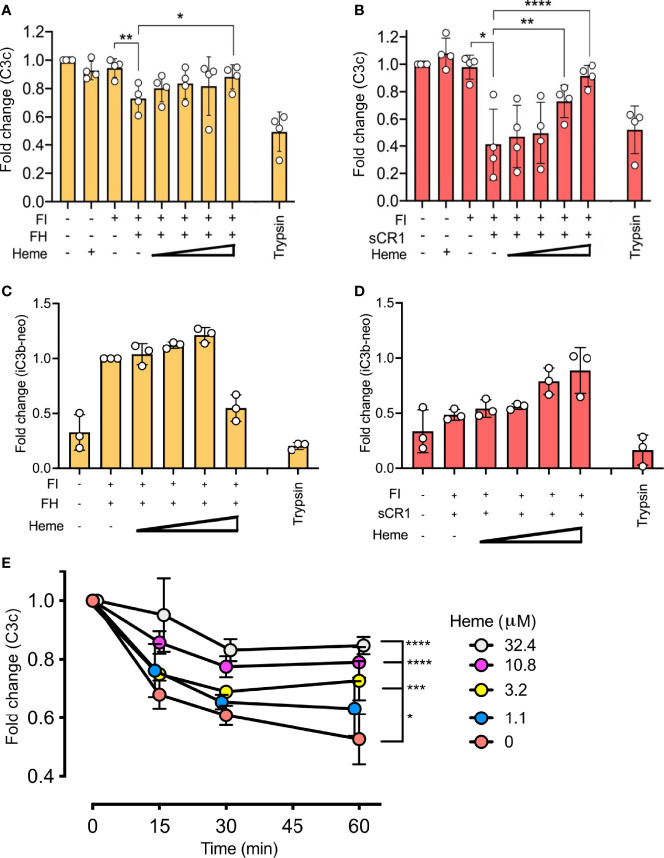
Effect of heme on factor I (FI)-mediated degradation of surface-bound C3b. C3b, covalently connected bound to magnetic beads, were analyzed for factor I (FI-mediated degradation by **(A–D)** purified components in phosphate buffered saline (PBS) and **(E)** lepirudin plasma at 37°C. Degradation of C3b was analyzed by a polyclonal anti-C3c antibody **(A, B, E)**, and a monoclonal iC3b antibody **(C, D)**. **(A–D)** Factor I (FI, 0.1 μg) and **(A, C)** factor H (FH, 0.1 μg) or **(B, D)** soluble complement receptor 1 (sCR1, 0.1 μg) were incubated with C3b-beads at 37° C for 60 minutes in PBS pH 7.4. Heme was supplemented in increasing amounts (1.1, 3.2, 10.8, 32.4 μM). Trypsin was included as a positive control. Values are shown as the mean of three independent experiments +/- standard deviation. Paired t-tests were used to compare C3b-beads and FI with and without co-factor. **(E)** One-way ANOVA non-parametric test was used for the comparison between C3b-beads with FI and co-factor with various concentrations of heme. **(E)** C3b-beads were incubated in lepirudin-plasma supplemented with compstatin Cp40 (8 μM) and heme in concentrations from 0–32.4 μM for 15, 30, or 60 minutes at 37°C. The graph shows mean +/- standard deviation from three independent experiments, all normalized to the mean fluorescent intensity (MFI) of beads at the start of incubation. One-way ANOVA non-parametric test was used for the comparison between C3b-beads with heme versus C3b-beads with various concentrations of heme. *p < 0.05, **p < 0.01, ***p < 0.001, ****p < 0.0001.

The impact of heme on factor I-activity was further evaluated by the degradation of surface-bound C3b. Magnetic beads with C3b coupled *via* the C3b-thioester were incubated with factor I and factor H (**Figures 3A, C**) or sCR1 (**Figures 3B, D**) at 37°C for 60 minutes. Heme was included from 32.4 μM and in threefold dilutions to 1.1 μM. The degradation of C3b was evaluated as reduction of C3c-fragment-detection (**Figures 3A, B**) or increased detection of iC3b on the beads (**Figures 3C, D**). Factor I, together with factor H or sCR1, was able to degrade C3b on the beads’ surface, as shown by decreased amounts of C3c on the bead’s surface (**Figures 3A, B**) and increased detection of iC3b (**Figures 3C, D**). The loss of C3c was more profound when sCR1 was used as a co-factor, and increase in iC3b was more profound for factor H. Heme interfered with this degradation in a dose-dependent manner, as more binding of the C3c-fragments was detected when heme was included in the incubations. Heme at 32.4 μM significantly (p < 0.01) reduced the loss of C3c-fragments from the beads compared to the conditions without heme. In separate experiments, the ability of heme to interfere with C3b-degradation in 20% lepirudin-plasma, containing only endogenous factor I and soluble co-factors; factor H and sCR1, was evaluated by incubating C3b-coated beads with increasing concentration of heme (up to 32.4 μM) for up to 60 minutes (**Figure 3E**). Incubation of the beads in plasma showed a time-dependent reduction of C3c-containing fragments on the bead surface. The addition of heme to plasma inhibited the C3b-degradation in a dose-dependent manner, and the effect was more pronounced with higher heme-concentrations. All tested heme-concentrations (1.1 – 32.4 μM) significantly (p < 0.05) reduced the liberation of C3c from the beads.

### Factor I in Plasma Is Encountered in Complex With Hemopexin

**Figure 4:**
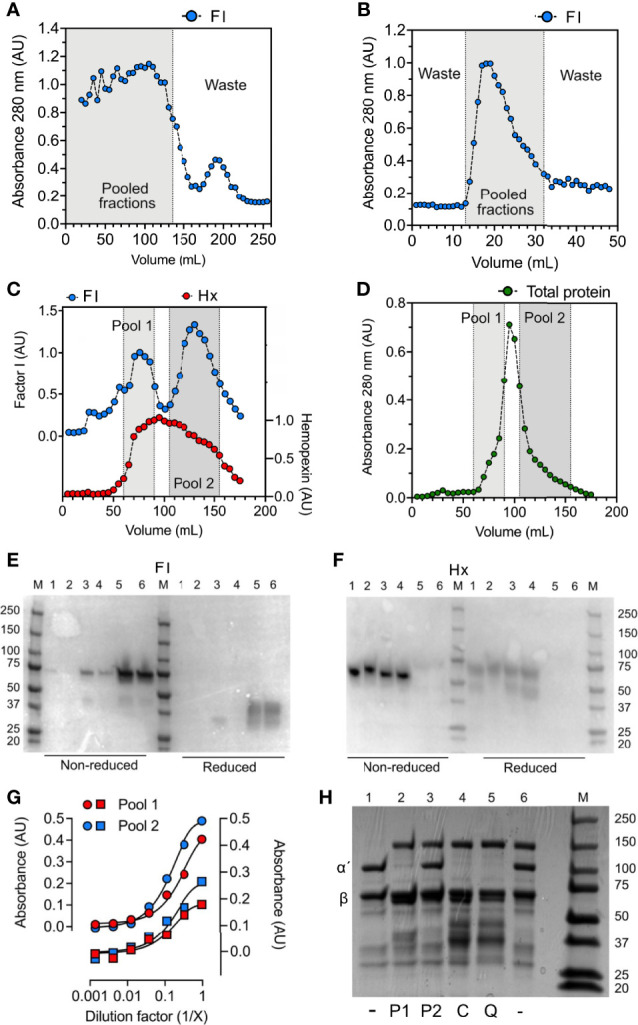
Hemopexin co-purifies with factor I. For the purification of factor I (FI), human plasma was, after PEG precipitation, centrifuged at 13 000 x g for 30 minutes, and the supernatant was applied for sequential chromatography. **(A–D)** Chromatograms for **(A)** Sepharose FF, **(B)** lectin agarose, and **(C, D)** Mono Q columns. **(A–C)** Fractions were analyzed by direct ELISA by coating fractions in microtiter plate wells and analyzing for factor I (FI, blue) and hemopexin (only the Mono Q column) (Hx, red) content using specific polyclonal antibodies. **(D)** Total protein in fractions from the Mono Q column was evaluated by absorbance measurements at 280 nm (absorbance units, AU). **(A, B)** Fractions within the gray area were pooled and applied for the next column. **(C, D)** The elution fractions from the Mono Q column **(C, D)** were pooled in two different pools: 1 (light grey) and 2 (dark grey) according to the two elution peaks for factor I **(E, F)** Western blot for factor I **(E)** and hemopexin **(F)** under non-reduced and reduced conditions. The following samples (10 μg) were analyzed: hemopexin purified from human serum (1), recombinant hemopexin (2), factor I “pool 2” (3), factor I “pool 1” (4), purified factor I bought from Quidel (5), purified factor I bought from CompTech (6). M corresponds to molecular weight marker. **(G)** Sandwich ELISA with pool 1 (red) and pool 2 (blue) incubated in threefold dilutions on microtiter plates coated with anti-FI (circles) and anti-hemopexin (squares) and detected with anti-hemopexin (circles) and anti-FI (squares). **(H)** SDS-PAGE for the degradation of C3b with different factor I-preparations (as indicated below the gel), incubated for 60 minutes at 37°C. All samples contain C3b, samples 2-6 contains factor H, and samples 2-5 contain factor I: pool 1 (P1) (2), pool 2 (P2) (3), CompTech factor I (C) (4), and Quidel factor I (Q) (5). The intact alpha´- and beta chain of C3b are indicated by α´ and β, respectively.

Factor I was purified from normal human citrate-plasma by sequential chromatography on a Sepharose FF column (**Figure 4A**), lectin agarose column (**Figure 4B**), and a Mono Q ion-exchange column (**Figures 4C, D**). From the latter, factor I was eluted in two peaks (**Figure 4C**). These peaks were pooled separately and referred to as pool 1 and pool 2 representing the early and late eluted peaks of factor I. By an enzyme-linked immunosorbent assay (ELISA) (**Figure 4C**) and Western blot (**Figures 4E, F**), hemopexin was identified as the main contaminant present in both pools. In comparison to pool 2, Pool 1 contained a lower amount of factor I (**Figure 4E**) but a higher amount of hemopexin (**Figure 4F**). Interestingly, factor I was present in low amounts in a hemopexin-preparation purified from normal human serum. No hemopexin was detected in the factor I-preparations bought from CompTech or Quidel (**Figure 4F**). Hemopexin-factor I complex formation was evaluated by sandwich-ELISAs where pool 1 was incubated in microtiter-wells coated with antibodies directed against factor I and detected with anti-hemopexin and vice versa (**Figure 4G**). Both assays showed that the eluate contained complexes consisting of both factor I and hemopexin. An effort to remove hemopexin from factor I in pool 1 by applying it to a heme-agarose column caused a complete loss of factor I (data not shown). Both pools were evaluated for their ability to degrade fluid phase C3b in the presence of co-factors. Only factor I in pool 1, and the commercial preparations were found to have catalytic activity, whereas no activity was found for factor I in pool 2 (**Figure 4H**).

### Patients With SCD Demonstrate Decreased Levels of Hemopexin and Factor I/Hemopexin Complexes

**Figure 5:**
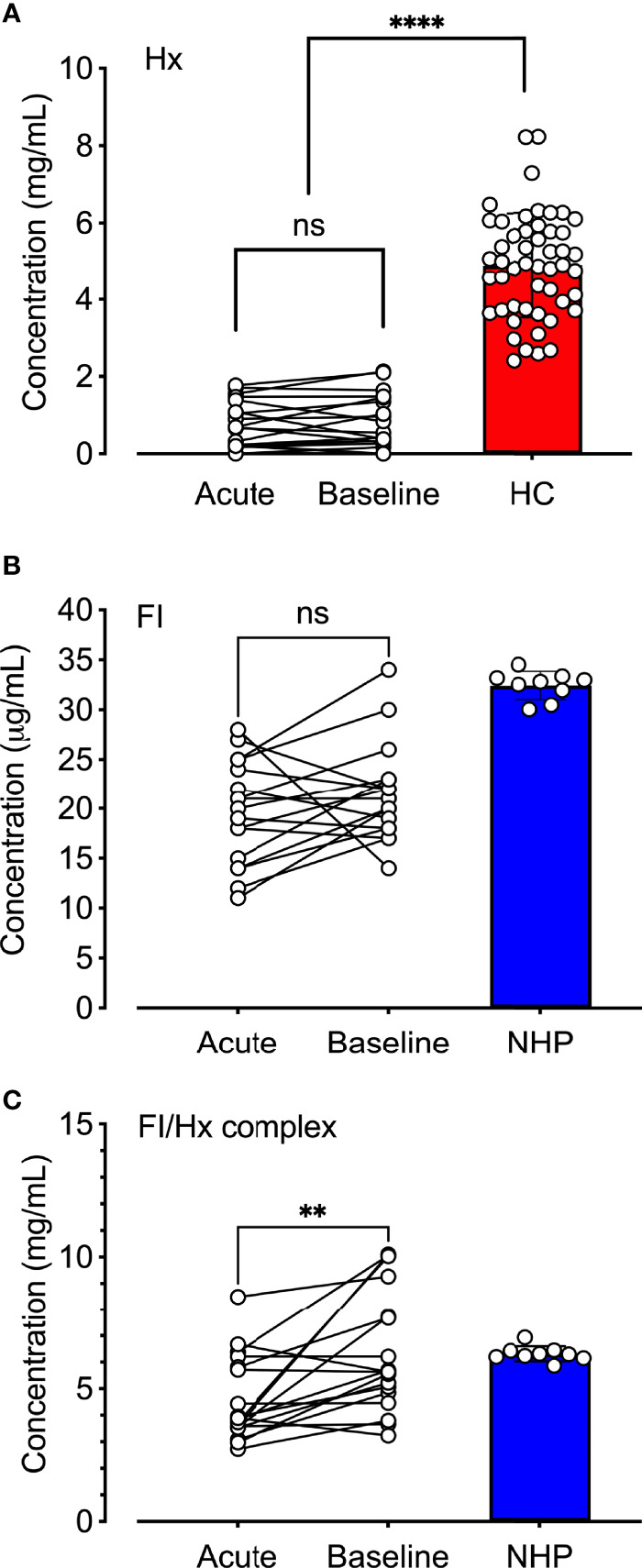
Quantification of hemopexin, factor I, and factor I-hemopexin-complexes in patients with sickle cell disease. **(A)** Hemopexin (Hx), **(B)** factor I (FI), and **(C)** factor I/hemopexin-complexes (FI-Hx complex) were quantified in EDTA-plasma samples from patients with SCD (n = 18) obtained during acute vasoocclusive pain crisis (acute), and a follow-up sample was collected at their baseline healthy visit at least four weeks after the acute episode (baseline). Normal human EDTA-plasma from 52 healthy individuals was used as controls for the hemopexin measurements (healthy controls; HC, red), and a pool of NHP (blue) sampled from six healthy donors was used as a control for the FI and the FI-Hx complex measurements. Individual values are shown together with mean and standard deviation. Paired t-test was performed in order to statistically compare the differences between the patients’ admission to the hospital and the healthy visit. One-way ANOVA was used for the comparison of patients at admission and baseline versus healthy donors. ns, non-significant, **p < 0.01, ****p < 0.0001.

The observation that hemopexin and factor I co-purified by two separate protocols (i.e., the factor I- and hemopexin purification protocol) led us to hypothesize that hemopexin might form a complex with factor I already in human plasma. Factor I, hemopexin, and factor I-hemopexin complexes were evaluated in normal human plasma, and patients with SCD (n = 18) collected during admission to the hospital during VOC and later during a baseline follow-up visit. Hemopexin levels were significantly lower (p < 0.0001) in the patients with SCD both during ‘acute’ and ‘baseline’ when compared to healthy controls (n=50) (**Figure 5A**). The mean hemopexin concentration (+/- SD) in this patient population increased from 0.80 +/- 0.59 mg/mL during an acute crisis to 0.86 mg/mL +/-0.69 during their baseline visits, although not statistically significant. The mean concentration of factor I was lower in patients with SCD in comparison to a pool of plasma samples from healthy donors (**Figure 5B**). The mean concentration (+/- SD) increased from 19.6 +/- 5.1 µg/mL to 21.3 +/- 4.8 µg/mL from admission to heathy visit, which was not statistically significant. Factor I-hemopexin complexes were found in the plasma pool from healthy donors, and in SCD patients (**Figure 5C**). Interestingly, these complex levels increased by 39% from ‘acute’ to ‘baseline’ visits (p < 0.01).

### Hemopexin Rescues Factor I-Activity

**Figure 6:**
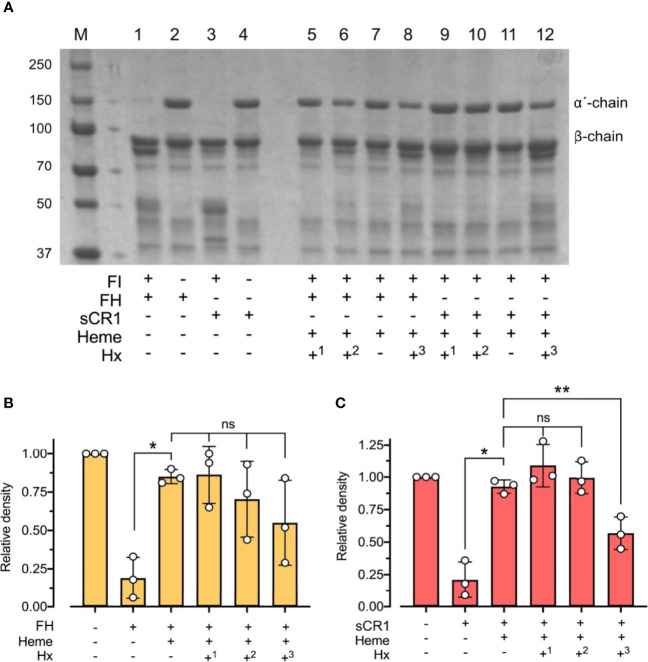
Effect of hemopexin in heme-dependent inhibition of factor I-activity. **(A)** C3b (10 μg) was incubated with factor I (FI 0.1 μg), factor H (FH 0.1 μg) or soluble complement receptor 1 (sCR1 0.1 μg), heme (32 μM), and hemopexin (Hx) added at different time points for 60 minutes at 37°C. The samples were run on a 4 - 15% SDS-PAGE under reduced conditions and stained with Coomassie Brilliant Blue, shown as a representative gel from three independent experiments. Sample 1 - 4 represent controls without heme or hemopexin. Sample 5 - 12 include heme and hemopexin (40 μg/ml) added at different time points. In samples 5 and 9, FI was preincubated with heme for 15 minutes before adding C3b, co-factor, and Hx (+^1^). In samples 6 - 7 and 10 - 11, all components were added simultaneously, but samples 7 and 10 lack Hx (+^2^/-). In samples 8 and 12, FI and Hx were preincubated for 15 minutes before adding C3b, heme, and FH or sCR1 (+^3^). **(B, C)** C3b-degradation was evaluated by densitometric measurement and displayed as the relative density of the C3b intact α´-chain at 101 kDa in relation to the β-chain at 75 kDa using factor H **(B)** and sCR1 **(C)** as the co-factors. The values are shown as mean +/- standard deviation of n = 3. Ordinary or repeated-measures one-way ANOVA with Dunnett’s multiple-comparison and paired t-tests for the comparison of two columns were used for the statistical analysis. ns, nonsignificant, *p < 0.05, **p < 0.01.

To evaluate the physiological role of hemopexin in the context of heme and factor I-mediated degradation of C3b, heme at 32 μM was incubated for 15 minutes at 37°C with C3b, factor I, and either factor H or sCR1, and with hemopexin added at different stages of the incubations (**Figure 6**). Pre-incubation of factor I with heme for 15 minutes before adding C3b, co-factor, and hemopexin led to heme-mediated inhibition of the C3b-degradation, despite the scavenger-property of hemopexin (**Figure 6**). In contrast, pre-incubation of factor I with hemopexin for 15 minutes and then adding heme rescued some factor I-activity, which resulted in partial degradation of C3b, indicating that hemopexin was able to protect factor I. This difference in C3b alpha´-chain degradation was seen for both co-factors and was statistically significant for sCR1 (p < 0.01) (**Figure 6**). When all the components were added simultaneously, heme inhibited C3b cleavage effectively even in the presence of hemopexin indicating that hemopexin could protect factor I from heme, but only if hemopexin was allowed to incubate with factor I before heme was introduced.

## Discussion

We found heme to interact with factor I, which interfered with the factor I-mediated degradation of C3b. Heme administered to either purified factor I and co-factors in buffer or lepirudin-anticoagulated plasma in concentrations corresponding to levels found in severe hemolytic conditions (2, 20, 40), impaired C3b-degradation of both soluble and surface-bound C3b. Our findings support a novel mechanism of how heme-dependent activation of the complement system depends on an impaired factor I-mediated degradation of C3b, leading to a dysregulation of the complement system, which promotes excessive C3-convertase formation and C3-cleavage.

Cell-free heme is a pro-inflammatory molecule recognized as a danger signal implicated in innate immune activation (14). Due to heme´s physicochemical properties, i.e., small hydrophobic and prooxidative (19, 41), heme can interact with several plasma proteins causing conformational changes and act as both an inhibitor (42) or inducer of inflammation (43). Several studies have investigated the role of complement activation in hemolysis and the liberation of heme. For example, in SCD, heme-induced complement activation has exclusively been attributed to alternative pathway activation (26–28, 44). Heme serves additionally as an inhibitor of the classical pathway by interacting with the C1q globular heads (37). However, the detailed mechanistic understanding for the strong heme-induced alternative pathway activation has been elusive.

*In silico* modeling showed that heme could interact directly with the native C3 close to the thioester domain, potentially mediating C3-hydrolysis (2). Hydrolysed C3, i.e., C3(H_2_O), serves as an initiator for complement activation by forming the fluid phase C3(H_2_O)Bb-convertase. The C3(H_2_O)Bb-convertase is, compared to the surface-bound C3bBb, a low-rate C3-convertase and cannot solely explain the strong activation seen from cell-free heme in plasma (45). However, C3(H_2_O)Bb may serve as an initiator of activation by generating the initial C3b molecules. Factor H serves as co-factor for the degradation of C3b to iC3b, and CR1 as co-factor for C3b to iC3b and further to C3c and C3dg. Nevertheless, factor H does not bind heme, and it is unlikely that heme will interfere directly with the factor H function (2).

Nevertheless, the interaction of heme with the C3b degrading enzyme factor I have not been previously investigated. Here we show that heme binds directly to factor I by spectroscopy and SPR, likely between the heavy and the light chain. Based on molecular modeling, we hypothesized that heme might impact the capacity of factor I to degrade C3b. To test this hypothesis, the degradation of soluble C3b was evaluated by loss of the intact 101 kDa a´-chain of C3b in SDS-PAGE and surface-bound C3b by increased expression of iC3b, as evaluated by a neoepitope specific antibody, and reduced binding of an anti-C3c antibody. We found that both co-factors could assist in the factor I-mediated degradation of both soluble and surface-bound C3b. There was a greater increase in iC3b-expression with factor H as the cofactor, and more significant reduction in C3c-detection on the beads by using sCR1 as the co-factor. This is most certainly explained by the sCR1-catalyzed cleavage of C3b all the way to C3dg with release of C3c-fragment into the fluid phase, whereas factor H can only catalyze cleavage of C3b to iC3b, which retains the C3c-fragment still on the beads (22). Heme-dependent inhibition of factor I catalytic activity was evident in the presence of either co-factor in the incubations. It occurred in plasma, where only endogenous soluble complement components were present. Heme as low as 1.1 μM, when added to plasma diluted 1:5, inhibited degradation of surface-bound C3b. This amount of heme can be readily observed in hemolytic diseases. All of these experiments were performed in the presence of the C3-inhibitor compstatin Cp40 to prevent additional C3b-deposition.

Hemopexin is the main scavenger molecule for the clearance of cell-free heme in plasma (14, 18). By coincidence, we found hemopexin to form a complex with factor I in plasma. Hemopexin was found to be the major contaminant when purifying factor I from human plasma whereas it was not found in the commercial factor I preparations. Factor I was eluted from the Mono Q column in two peaks, and hemopexin was present in both of these. Incubation with heme-agarose in order to remove hemopexin also completely depleted factor I from the solution. Correspondingly, factor I was identified in a hemopexin-preparation. To the best of our knowledge, this is the first time hemopexin and factor I are reported to circulate in complex. Surprisingly, we did not detect a direct interaction between FI and hemopexin when was analyzed by SPR. Heme did bind to FI and hemopexin but did not promote the interaction between the two proteins. It is possible that the conformation or orientation of the partners is altered upon the covalent binding to the chip, which could have obstruct potential direct interaction, or that complex formation requires a third component, other than heme. To further evaluate our findings, we tested our hypothesis using samples collected from patients with SCD during acute VOC, and later at their own baseline. The factor I-hemopexin-complexes were also present in low concentrations in the samples of these patients during VOC but increased to levels similar to normal plasma a few weeks after VOC, in contrast to the individual components, which remained low.

Administration of plasma-derived hemopexin before or during VOC in the Townes SCD mouse model, triggered either by heme or hypoxia-reoxygenation, have shown that administration of hemopexin can prevent or reduce VOC, but also, have antioxidant and anti-inflammatory effects (46, 47). Therefore, an objective became to investigate hemopexin’s role in the interplay between factor I and heme. When factor I was pre-incubated with heme, and hemopexin was added together with C3b, hemopexin could not rescue factor I activity. On the contrary, when hemopexin was pre-incubated with factor I, before exposure to heme, hemopexin to some extent rescued factor I’s activity, allowing more cleavage of C3b since we could partly observe the degradation of the alpha ´-chain.

Patients with SCD have a significant decrease in life expectancy compared to age-matched cohorts. If we consider their pain-adjusted quality of life, patients with SCD nearly have half the number of years compared to their counterparts. Those with more than four VOCs/year tend to have a shorter life expectancy (48, 49). Additionally, VOC has been recently associated with complement activation (50), and C5a infusion was shown to induce VOC in murine SCD models, an effect that could be blocked by targeting either C5a or C5aR (51). These results further highlight the importance of understanding the molecular mechanisms of action of hemolysis-derived heme in the pathophysiology of hemolytic diseases.

In conclusion, we propose a novel mechanism explaining how elevated levels of cell-free heme in the circulation interfere with the factor I-regulatory capacity of the complement system, mediating the inability to cleave C3b. These data have implications for all hemolytic diseases, where continuous or acute destruction of erythrocytes mediates initiation of complement activation. On the other hand, hemopexin seems to be a crucial antagonist of heme in preventing heme’s deleterious impact on factor I. In conclusion, the study offers new insight into the role of heme in the inflammatory process and may contribute to developing alternative therapeutic approaches for intravascular hemolysis.

## Data Availability Statement

The original contributions presented in the study are included in the article/**Supplementary Material**. Further inquiries can be directed to the corresponding author.

## Ethics Statement

The study was designed and performed according to the ethical guidelines from the declaration of Helsinki. Patients with sickle cell disease were recruited at Children’s Healthcare of Atlanta, Georgia, USA under a local IRB-approved study. Sampling of human whole blood from healthy individuals for preparation of plasma was approved by the ethical committee of the Norwegian Regional Health Authority, ethical permit REK#S-04114, 2010/934. Informed written consent was obtained from all blood donors. Written informed consent to participate in this study was provided by the participants’ legal guardian/next of kin.

## Author Contributions

AG designed and performed experiments and wrote the paper. JD, AZ, VP, VK, and KM performed or/and designed experiments. SC provided study material and edited the manuscript. KE, TM, and CM provided critical discussions and edited the manuscript. LR and PN designed and supervised the project, wrote the paper. All authors approved the final version of the manuscript.

## Funding

This work was supported by the Norwegian Research Council (Project 274332), the Swedish Research Council (Project 2018-04087 and 2016-04519), and the Crafoord Foundation (Project 20210961 and 20190890).

## Conflict of Interest

The authors declare that the research was conducted in the absence of any commercial or financial relationships that could be construed as a potential conflict of interest.

## Publisher’s Note

All claims expressed in this article are solely those of the authors and do not necessarily represent those of their affiliated organizations, or those of the publisher, the editors and the reviewers. Any product that may be evaluated in this article, or claim that may be made by its manufacturer, is not guaranteed or endorsed by the publisher.
