# Supplementary material for: Heme Interferes With Complement Factor I-Dependent Regulation by Enhancing Alternative Pathway Activation
Source: Front Immunol. 2022 Jul 22;13:901876. doi: 10.3389/fimmu.2022.901876 (PMC9354932; doi:10.3389/fimmu.2022.901876)
Supplement: Supplementary file 1 [file DataSheet_1.pdf]

Supplementary Material

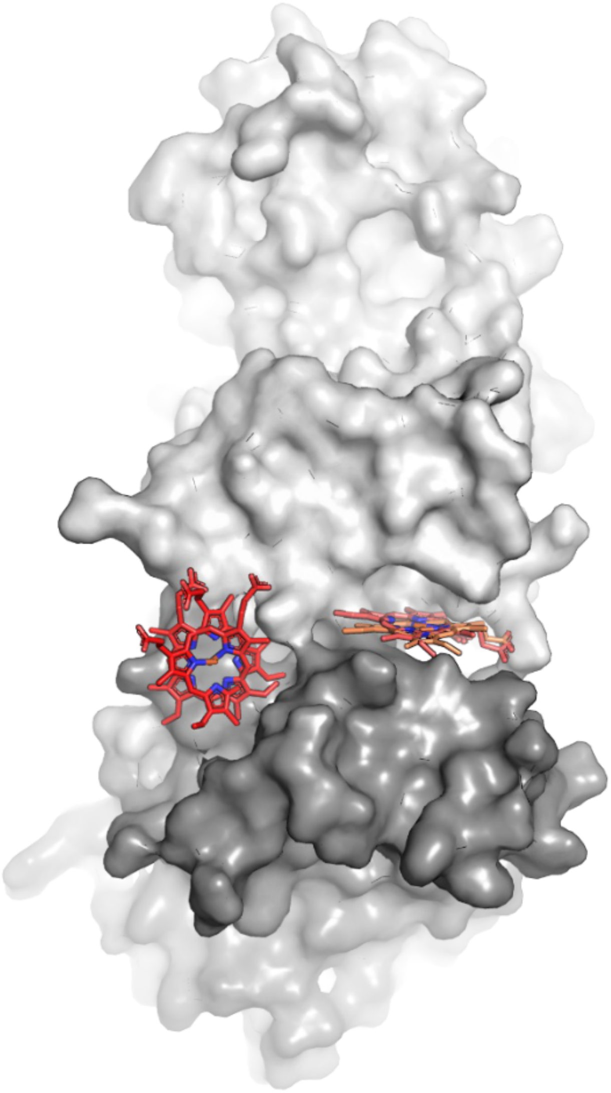

Supplementary Figure 1. Heme docking to factor I (PDB: 2XRC) predicts a location of the heme molecule at the interface between the heavy and light chain of factor I. The top ten solutions are displayed, with heme molecules from different solutions shown in shades of red. The heavy and light chains of factor I are shown in light and dark grey respectively.
